# A comprehensive platform for highly multiplexed mammalian functional genetic screens

**DOI:** 10.1186/1471-2164-12-213

**Published:** 2011-05-06

**Authors:** Troy Ketela, Lawrence E Heisler, Kevin R Brown, Ron Ammar, Dahlia Kasimer, Anuradha Surendra, Elke Ericson, Kim Blakely, Dina Karamboulas, Andrew M Smith, Tanja Durbic, Anthony Arnoldo, Kahlin Cheung-Ong, Judice LY Koh, Shuba Gopal, Glenn S Cowley, Xiaoping Yang, Jennifer K Grenier, Guri Giaever, David E Root, Jason Moffat, Corey Nislow

**Affiliations:** 1 Donnelly Centre and Banting & Best Department of Medical Research, University of Toronto, Toronto, Canada; 2 Department of Molecular Genetics, University of Toronto, Toronto, Canada; 3 Broad Institute, Cambridge, USA; 4 Department of Pharmaceutical Sciences, University of Toronto, Toronto, Canada

## Abstract

**Background:**

Genome-wide screening in human and mouse cells using RNA interference and open reading frame over-expression libraries is rapidly becoming a viable experimental approach for many research labs. There are a variety of gene expression modulation libraries commercially available, however, detailed and validated protocols as well as the reagents necessary for deconvolving genome-scale gene screens using these libraries are lacking. As a solution, we designed a comprehensive platform for highly multiplexed functional genetic screens in human, mouse and yeast cells using popular, commercially available gene modulation libraries. The Gene Modulation Array Platform (GMAP) is a single microarray-based detection solution for deconvolution of loss and gain-of-function pooled screens.

**Results:**

Experiments with specially constructed lentiviral-based plasmid pools containing ~78,000 shRNAs demonstrated that the GMAP is capable of deconvolving genome-wide shRNA "dropout" screens. Further experiments with a larger, ~90,000 shRNA pool demonstrate that equivalent results are obtained from plasmid pools and from genomic DNA derived from lentivirus infected cells. Parallel testing of large shRNA pools using GMAP and next-generation sequencing methods revealed that the two methods provide valid and complementary approaches to deconvolution of genome-wide shRNA screens. Additional experiments demonstrated that GMAP is equivalent to similar microarray-based products when used for deconvolution of open reading frame over-expression screens.

**Conclusion:**

Herein, we demonstrate four major applications for the GMAP resource, including deconvolution of pooled RNAi screens in cells with at least 90,000 distinct shRNAs. We also provide detailed methodologies for pooled shRNA screen readout using GMAP and compare next-generation sequencing to GMAP (i.e. microarray) based deconvolution methods.

## Background

Beginning in the late 1990's, construction of a systematic, barcoded gene deletion strain collection in *S. cerevisiae *set the stage for high throughput functional screening in eukaryotes [1,2]. Researchers using that resource developed powerful functional genomics screening approaches, and significantly, introduced the concept of performing complex, pooled population screens to simultaneously interrogate an organism's entire genome to identify drug targets, synthetic genetic interactions and other phenomena [1,3-7]. Similarly, the contemporary development of large libraries of short hairpin RNAs (shRNAs) and open reading frame (ORF) collections has significantly expanded the research toolkit available for performing mammalian functional genetics in a comprehensive manner [8-11]. The development of such tools, combined with the years of lessons from yeast-based screens suggested that similar genome-wide screens using a 'barcoded pool' screening strategy should be possible in a mammalian context [12,13]. Indeed, several groups have demonstrated the impressive ability of pooled RNAi dropout screens to identify genes essential to cancer cell proliferation [14-18]. Based on these early reports, pooled RNAi screens hold great promise for identifying genes important for growth, metabolism, differentiation, DNA damage response, sensitivity/resistance to therapeutics and many other processes in both normal and diseased cells.

Linking phenotypic changes to gene dosage in a heterogeneous population of cells requires a stably inherited molecular tag, such as a DNA barcode, that uniquely associates the phenotype with the perturbagen. A decade of work with yeast and other microbes has shown that DNA barcodes are sensitive and quantitative genetic markers that permit cell-based screening in high complexity pooled formats, with subsequent deconvolution by amplification of barcodes followed by microarray hybridization or high throughput DNA sequencing [19-21]. In contrast to the yeast knock-out strains, for pooled shRNAs and ORFs the growth perturbing agents themselves can serve as specific barcodes because they consist of uniquely identifiable DNA sequences. This approach has been demonstrated in several recent publications using different shRNA libraries [14-17]. For each publication, a different custom array was built and quality controlled, but information on the optimization and validation was limited, and the microarrays were not made commercially available. Consequently, researchers interested in utilizing this technology would need to perform resource intensive optimization and quality assurance on a new custom microarray for each shRNA library - an approach that is cost-inefficient, and does not promote standardized datasets amenable to high level informatics analysis. In order to facilitate loss-of-function and gain-of-function pooled screens using commercially available gene modulation libraries, we designed and validated a single microarray detection platform for deconvolution of loss- and gain-of-function pooled screens in human, mouse and yeast cells.

One can now easily procure custom microarrays designed to detect novel barcoded libraries. In order to evaluate and maximize the effectiveness of such barcoded platforms, extensive testing and optimization is required. For gene expression studies, extensive development work has already been done for a number of detection platforms and, as a result, they have become straightforward and routine to use. For new custom barcoded systems, similarly rigorous testing, optimization of protocols, and development of quality control standards must be done. This manuscript describes a detailed evaluation and analysis of the performance of the Gene Modulation Array Platform (GMAP) microarray, and carefully optimized protocols for every step of the process from sample preparation to data analysis. In essence, we provide a guide to use the GMAP chip as well as highlight the design principles and quality control measures that may be useful for other customized microarray platforms. In addition, we have also developed a software tool (using open source scripts) for use in extracting and analysing the data from the GMAP.

## Results and Discussion

We designed the multipurpose, cost-effective GMAP to enable researchers to inexpensively and comprehensively collect data from genome-scale pooled gene-dosage modulation screens performed in human, mouse, and yeast cells using commercially available gene modulation libraries on a standard platform. Specifically, the GMAP enables readout of clone enrichments and depletions from pooled screens using the RNAi consortium (TRC) human and mouse libraries [11,14,22], human ORF expression pools [23,24], and pooled screens using gene deletion-associated barcodes or ORFs from budding yeast[1,25].

The detection features on GMAP are summarized in Table 1. The GMAP features encompass the entire TRC1 shRNA human and mouse collections and a portion of the greatly expanded TRC2 collection such that >248,000 unique shRNAs from the TRC collection can potentially be assayed in parallel. To achieve this extraordinary density, GMAP features were synthesized at a 5 μm scale, resulting in a 4.84-fold smaller surface area than the 11 μm features on the TRCBC array, a commercially unavailable microarray that was previously generated to perform pooled shRNA screens on a subset of the TRC1 shRNA collection [14]. We based the design of the GMAP shRNA features on observations from the TRCBC microarray which included three different, but highly overlapping sequences to detect each shRNA[14]. One of these three feature designs was superior to the others in tests using engineered pools with known relative shRNA compositions (Additional file 1, Figure S1). The GMAP chip starts with the best performing feature design and adds one base from the 21-base shRNA stem sequence to extend the feature length from 21 to 22 bases. Three identical replicates per shRNA barcode feature were included on the GMAP microarray. This strategy was used to maximize signal-to-noise and to identify and potentially correct for any subtle shRNA processing inconsistencies without significantly impacting specificity.

**Table 1:** Description of the features on the UT GMAP 1.0

Probe ID	Description	Unique probes	Replicates	Total number of probes	Probe length
hORF	Human ORFeome	134901	1	134901	25
huORF	HuGene ORFs	58087	1	58087	25
HP	shRNA sequences (mouse and human)	248049	3	744147	22
HPC	shRNA negative controls	138	33	4554	22
HSPI	Hybridization spike-in probes	200	25	5000	22
HPTMM	shRNA mismatch control probes	8097	3	24291	22
TAG	Yeast barcode probes	26801	3	80403	20
yORF	Yeast open reading frames	11421	1	11421	25
**Total**	**NA**	**487694**	**NA**	**1062804**	**NA**

In order to achieve consistent results, a simple-to-follow standard operating procedure (SOP) was developed for preparing samples for hybridization (see Additional file 2 for detailed protocols and recipes). As part of the SOP optimization process, a standardized amount of shRNA probe was hybridized to the GMAP array. Hybridizations were performed with quantities of 1 μg, 2 μg, and 3 μg of purified shRNA probe (see **Materials and Methods**) that was generated from a plasmid pool of 78,432 (78 k) human shRNAs (Additional file 1, Figure S2a). 2 μg of probe gave the best signal-to-noise ratio and was therefore chosen as the standard quantity for all 78 k pool hybridizations. For pools of different complexity, the amount of probe applied to GMAP is altered proportionally compared to the 2 μg used for 78 k pools (for example ~1.35 μg for 54 k pools). In addition to optimizing the quantity of probe, a range of hybridization and washing temperatures were tested using 2 μg of 78 k shRNA pool probe. Hybridization was tested at 40°C and 45°C, with washing tested at 30°C and 35°C. Hybridization at 40°C, with array washing at 30°C was found to provide the highest probe signal with minimal signal from features corresponding to shRNAs not included in the pool (Additional file 1, Figure S2b).

**Figure 1:**
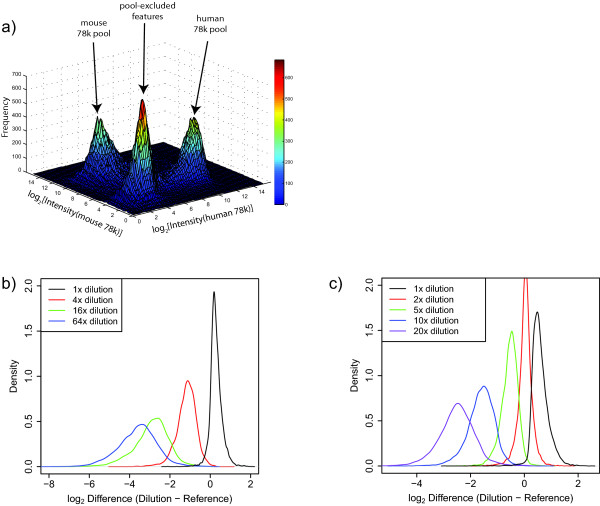
**Readout of high complexity shRNA pools**. **(a) **Surface plot of signal intensities for all 248,049 shRNA features on the GMAP microarray following hybridization of probe generated from the human 78 k plasmid shRNA pool or the mouse 78 k plasmid shRNA pool. The surface plot shows the signal intensity distributions of the human and mouse 78 k features plotted against each other and the remaining shRNA probe features not corresponding to either pool. The signal peaks are labelled according to which set of features they represent. **(b) **Distribution plots of GMAP features data for a dilution series of shRNAs in separate pools. 4x, 16x, and 64x curves are plotted as the distribution of log_2 _difference between the array signal for shRNAs in the dilution series and their signal in the reference (Even) pool. The 1x curve is plotted as the log_2 _difference between a group of undiluted shRNAs in the 4x pool and the same shRNAs in the Even pool. **(c) **Distribution plots of GMAP array data for a dilution series of shRNAs within the same pool. 2x, 5x, 10x, and 20x curves are plotted as the distribution of log_2 _difference between the array signal for groups of diluted shRNAs and their signal in the reference (Even) pool. The 1x curve is plotted as the log_2 _difference between a group of undiluted shRNAs in the 2x-20x pool and the same shRNAs in the Even pool. All microarrays were run in triplicate, and the replicate signal intensities were averaged.

To assess GMAP chip performance with complex shRNA populations, probe generated from the 78 k human shRNA pool or a different 78 k mouse shRNA pool was hybridized and analyzed. Signal intensity histograms and cumulative distribution plots generated from the collected data indicate that the signal intensities for probes corresponding to shRNAs present in the 78 k pools were well resolved from the background signal of GMAP features corresponding to shRNAs not present in the pools (Additional file 1, Figure S3a,b). The small overlap between the signals for features matched to either of the human or mouse shRNA pools versus the features that have no matching shRNA in the corresponding pools indicates low rates of library composition errors and cross-hybridization. A surface plot of intensities for all shRNA features on the GMAP array for the human 78 k pool versus the mouse 78 k pool indicates that large pools of equal complexity can be easily resolved from each other (Spearman correlation R < 0.01) and from shRNA features not corresponding to either experimental pool (Figure 1a).

One major application of pooled shRNA screens is to perform negative selection screens, so called "dropout screens", in cancer cell lines to identify cancer-essential genes that represent potential therapeutic targets. To assess the performance of the GMAP in deconvolving genome-scale shRNA drop-out screens, we first calculated a minimum signal in the reference data for inclusion in analysis. This was accomplished by adding 1.96 standard deviations (the 95% confidence limit) to the mean background log signal measured on human 78 k pool hybridizations, yielding a threshold of log_2 _= 7.89 (Additional file 1, Figure S3c). Consequently, 89.9% of the shRNA signals in the reference data exceeded this threshold and were retained for analysis. We simulated shRNA dropout screens by altering the concentration of sub-fractions of the human 78 k pool in two different shRNA dilution experiments. The first approach utilized four different 78 k human pools, each containing the same 78,432 shRNAs. Approximately 70,100 shRNAs were present in equal concentration in all four pools, while an identical sub-fraction of ~8,300 shRNAs were either undiluted (the reference "Even pool"), or diluted 4-fold, 16-fold, or 64-fold (the "4x", "16x" and "64x" pools" respectively). Distribution plots of shRNA log_2 _signal intensity difference between the diluted pools and the Even pool demonstrate that the diluted subpools can be distinctly resolved from each other (Figure 1b). In a second approach, we constructed a single 78 k shRNA pool (the "2x-20x" pool) in which, relative to the bulk population, four distinct subsets of ~8400 shRNAs each were diluted 2-fold, 5-fold, 10-fold and 20-fold, respectively. Similar to the previous experiment, distribution plots of shRNA signal difference between diluted pools and the reference population in the Even pool demonstrate that the diluted subpools are resolvable (Figure 1c). We did observe that the signal reduction in diluted subpools on the GMAP chip is not directly proportional to the dilution factor, indicating that changes in microarray signal are not linearly proportional to shRNA concentration across the full observed signal range. In particular, the subpool dilution signals are spaced more closely, or compressed, than would be expected for a linear concentration versus signal relationship.

To assess the performance of GMAP relative to the lower density and previous generation TRCBC array, we amplified shRNA probes from genomic DNA extracted from human BT-474 cells infected with a ~54,000 shRNA pool of lentiviruses corresponding to all available human shRNA features on the TRCBC chip, divided the probe and hybridized samples to both TRCBC and GMAP chips. Comparison of signal intensity between the two different array formats revealed a Spearman correlation coefficient of R = 0.96 (Additional file 1, Figure S4). This result confirms that moving from 11 μm feature sizes to 5 μm feature sizes does not cause any significant reduction in signal strength or specificity, agreeing with reported observations on yeast barcode arrays [26].

**Figure 2:**
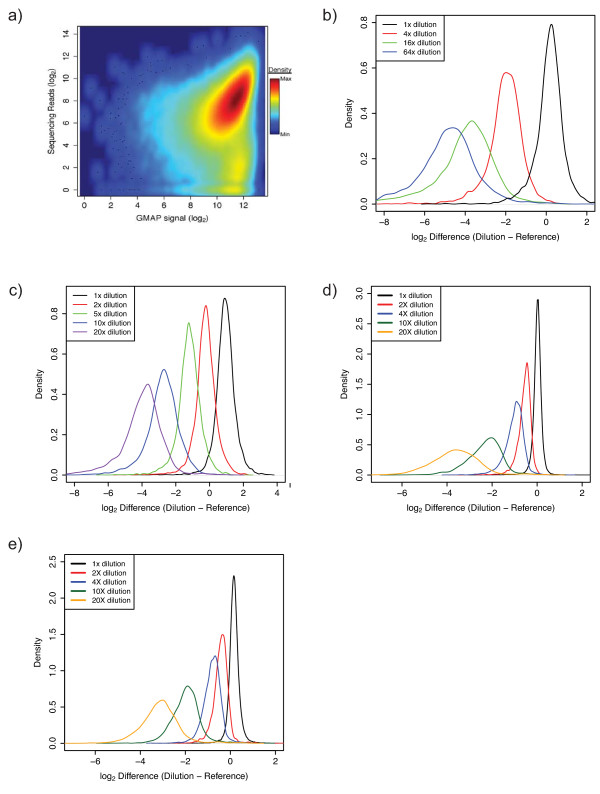
**Comparison of sequencing and GMAP performance with high complexity shRNA pools**. **(a) **Scatter plot of GMAP array signal intensity (X-axis) versus sequencing read number (Y-axis) for shRNA clones from the human Even shRNA pool. **(b) **Distribution plots of Illumina sequencing data for a dilution series of shRNAs in separate pools. 4x, 16x, and 64x curves are plotted as the distribution of log_2 _difference between the number of sequencing reads for shRNAs in the dilution series and their read count in the reference (Even) pool. The 1x curve is plotted as the log_2 _difference between a group of undiluted shRNAs in the 4x pool and the same shRNAs in the Even pool. **(c) **Distribution plots of Illumina sequencing data for a dilution series of shRNAs within the same pool. 2x, 5x, 10x, and 20x curves are plotted as the distribution of log_2 _difference between the number of sequencing reads for groups of diluted shRNAs and their read count in the reference (Even) pool. The 1x curve is plotted as the log_2 _difference between a group of undiluted shRNAs in the 2x-20x pool and the same shRNAs in the Even pool. **(d) **Distribution plots of GMAP features data for a dilution series of shRNAs contained within sub fractions of a ~90,000 shRNA pool where the probe was amplified from shRNA plasmid template DNA. **(e) **Distribution plots of GMAP features data for a dilution series of shRNAs contained within sub fractions of a ~90,000 shRNA pool where the probe was amplified from genomic DNA of A549 cells infected with lentiviral pools. 2x, 5x, 10x, and 20x curves are plotted as the distribution of log_2 _difference between the array signal for groups of diluted shRNAs in the 90 k Dilution pool and their signal in the 90 k Reference pool. The 1x curve is plotted as the log_2 _difference between a group of undiluted shRNAs in the 90 k Dilution pool and the same shRNAs in the 90 k Reference pool.

To compare the BAR-seq strategy [20] with microarray detection for shRNA barcodes, we used Illumina next-generation sequencing technology to enumerate barcodes in the same five human shRNA 78 k pools described above (Even, 4x, 16x, 64x and 2x-20x). Sequencing libraries were prepared from these pools via essentially the same approach used for generating labelled shRNA barcodes for the GMAP array, followed by an additional amplification step to incorporate adapter regions (see **Materials and Methods)**. Between 22.3 million and 25.6 million mapped sequence reads for each of the five shRNA plasmid pools were obtained, yielding a median number of reads per shRNA per 78 k pool of between 107 and 162 (Table 2). Notably, 73,073 (93.4%) of expected shRNA sequences were detected in the combined data from all five shRNA pools sequenced (>121 million mapped reads) and 68,420 (87.5%) of expected shRNA barcodes were detected in the human 78 k Even pool alone (Table 2). Comparison of the sequence read frequency to GMAP intensity signal for shRNA barcodes in the Even pool resulted in a positive Spearman correlation of R = 0.37 (Figure 2a). By omitting shRNA clones that were not enumerated in the sequencing data by at least 16 reads and with a signal intensity of at least log_2 _= 7.89 on the GMAP array, the correlation improved to 0.42. While both the sequencing and the chip hybridization methods provide an assessment of shRNA relative concentration that is reproducible, the modest level of correlation between the signals for these two types or readouts indicates that they have significantly different relationships to absolute shRNA concentration.

**Table 2:** Summary of shRNA pool deconvolution by next-generation sequencing

Statistic	Even	4x	16x	64x	2-20x
Reads passed filter	22407757	24596943	24793754	24304519	25684516
Mapped reads	22296083	24504006	24687271	24235485	25605983
Unmapped reads	111674	94937	106483	69034	78533
Unmapped reads %	0.5	0.39	0.43	0.28	0.31
1^st ^quartile	40	34	26	35	21
Median	153	149	140	162	107
Mean	315	352.4	351.6	350.7	370.2
3^rd ^quartile	375	408	404	417	385
Max	17960	19360	21750	20000	29460
Uncounted	9784	10748	10045	10983	10739

A major concern in using large shRNA pools with microarray detection strategies is the extent to which cross-hybridization occurs. To address this issue, we measured the frequency of significant signals from GMAP shRNA features that did not correspond to constituents of a pool of shRNA probes. Specifically, we examined features corresponding to the 78 k mouse pool for signal when the 78 k human pool was hybridized. Features on GMAP that had 100% identity to shRNAs in both the mouse and human pools were removed from the analysis. After doing so, we found that only 2637 of 77,690 (3.39%) mouse 78 k pool features had significant signal (log_2_≥7.89). From this finding, we infer that amongst the human shRNA features, the cross-hybridization rate from human 78 k pooled probe would be similar.

Frequency distribution plots of sequencing read counts for the Even, 4x, 16x, 64x and 2-20x pools (Figure 2b,c) show similar characteristics to the distributions generated for the same pools by microarray detection on the GMAP (Figure 1b,c) with some exceptions. First, the distributions tended to be slightly wider for sequence data. Second, the distributions for sequence data exhibit a linear relationship that more accurately reflects the actual experimental dilution of shRNAs in the dilution pools. In other words, the distribution curves for sequencing data tend to center over the correct fold-dilution. These observations demonstrate that sequencing barcode pools results in linear, quantifiable signals whereas microarray detection displays nonlinear signal, a behaviour previously observed by Pierce *et al. *[27]. In addition, the dynamic range of signal obtained from GMAP is compressed relative to deep sequencing (Additional file 1, Figure S5). However, these differences aside, overall, GMAP detection of shRNA sequences was similar to sequencing-based deconvolution in its sensitivity and quantitative reproducibility over a range of shRNA concentrations.

Microarray experiments have historically suffered from subtle to substantial differences in data produced from the same or similar templates when performed on different dates, by different users, or in different locations [28]. A candidate method to enable detection and/or correction of these differences is to include a standard set of synthetic oligonucleotide probes that are "spiked" into array hybridization mixtures in defined amounts. These hairpin spike-in (HPSI) probes, designed to have identical length and similar sequence characteristics to shRNAs, may be used as yardsticks for artifact detection and data normalization methods. We replicated 25 clusters of 200 HSPI features across the GMAP (Table 1). Spatially localized signal intensity fluctuations between HSPI clusters may indicate potentially poor hybridization or washing performance, contamination, or physical damage to the array surface. As a trial, 12 HSPI probes were tested in replicate array hybridizations to examine their hybridization characteristics and it was found that they behaved in a dose-dependent manner similar to labelled shRNA probes (Additional file 1, Figure S6).

To examine GMAP performance in a simulated cell screening experiment, two large pools of 90,408 shRNAs, each targeting ~18,000 human genes were constructed. For the "90 k Reference" pool, all of the shRNA plasmids were combined at approximately equal concentrations, while a dilution series pool, the "90 k Dilution", contained four sub-sets of ~3,500 shRNAs each that were diluted 2-fold, 4-fold, 10-fold or 20-fold with respect to their counterparts in the 90 k Reference pool. Distribution plots of shRNA log_2 _signal difference between the 90 k Dilution and the 90 k Reference pools demonstrate that the diluted sub fractions are clearly resolvable (Figure 2d). We generated lentivirus from the 90 k Reference and 90 k Dilution pools that was subsequently used to infect A549 cells. Genomic DNA prepared from these cells containing integrated shRNA-expressing cassettes was used as template for probe generation and GMAP hybridization. The resulting distribution plots of log_2 _signal difference between the 90 k Dilution and 90 k Reference pools post-infection (Figure 2e) were very similar to those achieved with probe generated from plasmid template for the 90 k pools and 78 k pools. A scatter plot comparing data for the 90 k Reference pool derived from plasmid and genomic DNA template reveals excellent correlation (R > 0.97, see Additional file 1, Figure S7) further demonstrating that reliable results can be obtained from pooled cell-based screening experiments with shRNA pools spanning essentially the entire human genome with coverage of 4-5 shRNAs per gene.

**Figure 3:**
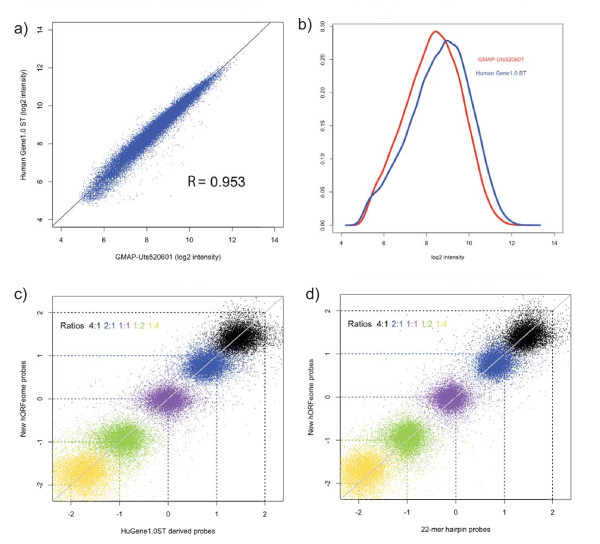
**Quality of human ORF features on GMAP chip**. **(a) **Intensity signals from the GMAP array (X-axis) plotted against signals from the Human Gene 1.0 ST array (Y-axis) following amplification of human ORFeome v5.1 pools and hybridization of the resulting probe to each of these arrays. Common features between the two arrays were used to calculate the Pearson correlation coefficient. **(b) **Distribution of signal intensities from replicate GMAP or Human 1.0 ST arrays as described in **(a) **for shared features. Hybridization behaviour for the huORF features compared to the **(c) **huGene features or the **(d) **22-mer hairpin features for corresponding genes.

To enable pooled ORF over-expression screening using the GMAP array to detect and quantify ORF sequences, we designed features against 22,449 distinct human ORF sequences in the Mammalian Genome Collection (MGC) (Table 1 and [29,30]). Between 1 and 8 probes were designed against each ORF, with a median of 7 probes per sequence. For comparative purposes, we also included up to three additional features found on the human expression profiling Affymetrix Gene 1.0 ST array for 18,088 of these sequences. To assess the GMAP performance with human ORF hybridization, we developed 41 plasmid pools of entry clones (15,347 open reading frames) from the human ORFeome v5.1 collection that were combined to generate a single master pool. Subsequently, 15,347 ORFs were amplified in pooled format with common flanking primers, labelled and hybridized to both the Human Gene 1.0 ST array and GMAP arrays in duplicate (see **Materials and Methods**). Signal for features shared between the two arrays was highly correlated (Pearson's correlation coefficient R = 0.953, Figure 3a), with similar distribution of signal across the features for each array (Figure 3b) and similar signal-to-noise ratios (data not shown). These results demonstrate that the GMAP has robust reporting of ORF data, and suggests that the GMAP can be used for a number of human gene assays including human ORF/cDNA overexpression screens.

To compare the dynamic range of signal for huORF and huGene features on the GMAP, five different quantities of probe generated from ORFeome plasmid pools were hybridized to GMAP in duplicate. The resulting data indicated that two-fold changes in probe input produced highly correlated signals across a 16-fold dynamic range (Figure 3c), thus we combined huORF and huGene features into one feature set. Our methodology to detect ORF sequences on microarrays depends on common flanking primers that amplify entire open reading frames. This provides an opportunity to examine the utility of the three sets of ORF features on the GMAP microarray, including the 22-mer hairpin features designed to detect shRNA barcodes. The signal concordance between sequence identity-conserved hORF features and shRNA features on GMAP was interchangeable across the entire spectrum of probe concentrations (Figure 3d). This concordance can be further exploited to expand the feature set for each gene to obtain more accurate measurements of ORFs in a given hybridization mix, which is particularly important for discriminating isoforms in a polyclonal library of open reading frames.

GMAP also contains triplicate copies of features for the collection of ~16,000 20 nucleotide Yeast Knockout Collection barcodes[2], and single copies of ~12,000 25 nucleotide yeast ORF-specific features (Table 1). The yeast ORFs represented on this array are identical to those found on the Tag4 barcode array with two distinct probes designed against each of 5718 yeast ORF sequences. These features can serve as additional controls as they are non-homologous to the shRNA or human ORFeome probes on the GMAP. The yeast features on the GMAP display comparable performance to the TAG4 microarray (Affymetrix) from which they are derived (data not shown).

A major hurdle to using a new microarray platform is the informatics associated with data extraction and analysis. The GMAP chip contains several subsets of features, some of which can be organized as traditional Affymetrix feature sets as well as features for interrogation of short input DNA sequences (shRNA barcodes). Extraction of signal from these arrays can be done with the Affymetrix Power Tool (APT) collection. To help aid adoption of the GMAP, we have developed a Java application to allow data reorganization, graphical summaries and downstream analysis of the datasets extracted from these arrays (files and applications available online at http://chemogenomics.med.utoronto.ca/supplemental/gmap/). This application makes use of a variety of R and Bioconductor libraries (see **Materials and Methods)**. The R functions that we have developed for this analysis will also be available as scripts/libraries to allow end users to work directly in the R environment. The current functionality of the application includes methods to extract signals into a single tab-delimited file for subsets of features and GC-matched background signal, normalization procedures, routines to generate signal ratios against one or more reference chips with options to fine-tune analyses and standard or user-defined annotation files for merging analyzed signal.

## Conclusions

Our goal was to develop a comprehensive and standardized microarray platform for quantification of pooled screening results using commercially available gene expression modulation libraries for human, mouse and budding yeast cells. The advantages of a standardized microarray are many-fold. First, a highly validated microarray is invaluable to research labs that want to adopt compatible screening approaches but do not have extensive resources to build, test and optimize a custom barcode microarray themselves. The GMAP presents an accessible solution because the obstacle of designing, constructing, and testing of the array as well as development of rigorous SOPs has already been overcome. Second, screening costs are reduced since the GMAP supports multiple commercially available functional screening approaches in a single solution. As such, individual labs or facilities do not have to re-optimize hybridization conditions for different microarray platforms, and a single framework can be used for data extraction and quality control analysis. Third, a standard microarray permits use of uniform methods across the many laboratories using these gene modulation libraries. Lastly, a multipurpose array can serve as a useful validation tool as new technologies for detecting barcodes utilizing deep sequencing mature and become more widely used. To enable facile adoption of the GMAP array for large-scale screen deconvolution, we have also provided a series of supplemental protocols and software tools to help extract data from functional screens using commercially available gene modulation libraries.

A thorough comparison of barcode detection strategies for large, complex shRNA pools has been lacking in the literature. We undertook such a comparison with pools comprised of ~78,000 and ~90,000 distinct lentiviral shRNAs, the largest such shRNA screening pools described to date. We directly compared microarray detection with deconvolution by Illumina deep sequencing and conclude that, at least in the current context, data quality is comparable using either method. For many or most users, the array-based read-out is currently more cost-effective on a per-sample basis when costs per sample are tabulated. Equally important as cost for many researchers is the turn-around time required for sample preparation, sample run time and data acquisition/processing, which is currently accomplished more rapidly using microarrays. Sample run time on the GMAP chip spans ~18 hours between probe application, hybridization, washing and scanning. Typical microarray platform equipped labs or core facilities should be able to easily accommodate more than 30 experimental samples in a typical work-week.

In addition to examining a plasmid pool containing >78,000 shRNA clones, we also simulated a cell-based dropout screen with a larger pool of >90,000 shRNA clones. Genomic DNA was isolated from each of the transduced populations and shRNA barcodes were detected using the GMAP. Importantly, the resolution of barcode detection was nearly equivalent whether the starting template was from plasmid DNA or genomic DNA. This experiment demonstrates that with the protocols developed here, the GMAP offers an effective and efficient means of screening very large pools in a cellular context. Our trials with complex shRNA and human ORF pools demonstrate that the GMAP array is capable of efficiently quantifying human and mouse genome-scale gene modulation screens. As well, the GMAP chip detection performance is equally suited for quantifying shRNA abundance in lower complexity pools. The GMAP array is not limited to use with the pooled reagents described here - in fact, any screen which uses the yeast deletion barcodes or TRC shRNA sequences as molecular barcodes can be analyzed using the GMAP array. Further generations of GMAP, utilizing even higher feature-count microarrays, could be expanded to include other shRNA and/or ORF libraries. However, benefits of such an expansion would have to be weighed against the increased cost of such expanded arrays. Generating specific arrays corresponding to different shRNA libraries might be more efficient and cost effective. In summary, this cost-effective platform provides any academic laboratory with access to a standardized array and a suite of methods and analytical tools to perform systematic genome-scale genetics on mammalian cells.

## Materials and methods

### Cell lines and growth conditions

BT-474 and A549 cells were obtained from ATCC and maintained in DMEM (Wisent Inc.) + 10% FBS (Life Technologies-Invitrogen) at 37°C and 5% CO_2_.

### HSPI probe design

Spike-in sequence-feature combinations were selected so that their perfect match Tm profiles (computed by MELTING[31]) would be similar to shRNA-feature combinations, but any mismatch Tm profiles would be significantly lower than the lowest shRNA-feature Tm profile. We computed Tm only for instances in which spike-in sequences had at least 13 bases of sequence identity to known shRNA features. Sequences could share 13 bases of identity anywhere within the designed 21-mer. Candidate sequences with fewer than 13 bases of identical sequence to any shRNA feature sequence were retained without Tm evaluation as unlikely to cross-hybridize, and candidate sequences with more than 19 bases of sequence similarity were eliminated outright without evaluating Tm. The top 200 sequences were selected for inclusion as spike-in features on the GMAP array.

### Pooled libraries

All shRNA pools and constructs in this study were derived from the RNAi Consortium lentiviral libraries [11,14,22] available from Sigma-Aldrich and ThermoFisher-Open Biosystems. The human and mouse 78 k shRNA lentivirus plasmid pools were assembled by combining equal proportions of ten sub-pools of ~8,000 clones each targeting either ~78,000 human or ~78,000 mouse transcripts. Dilution pools were constructed by the same method, except that diluted sub-pool fractions were reduced in their representation in the 78 k pool by the indicated amounts. Lentivirus pools were generated from pooled lentiviral plasmid DNA. 90 k plasmid pools were constructed by combining ~3500-4000 member shRNA sub-pools together. These sub-pools consisted of concentration-normalized plasmid. Dilutions were prepared as described for 78 k pools. The human ORF pool was derived from the human ORFeome v5.1 clones [23,24] available through ThermoFisher-Open Biosystems. 41 subpools of plasmid DNA were generated from 15,347 entry clones where each subpool contained ~380 human ORFs. A master hORF pool was derived from equivalent amounts of each of the 41 subpools normalized for the number of hORFs in each subpool.

### Pooled lentiviral infections

7 × 10^7 ^A549 cells per replicate were infected with either 90 k Reference or 90 k Dilution lentiviral shRNA pools at an MOI of 0.3-0.4. After four days of selection in puromycin-containing medium to eliminate uninfected cells, genomic DNA was prepared from shRNA-containing cell populations (Blood Maxi prep kit, Qiagen). 7 × 10^7 ^BT474 cells were infected with the human shRNA pool at an MOI of 0.3-04. After two days of selection in puromycin-containing medium to eliminate uninfected cells, genomic DNA was prepared from shRNA-containing cell populations.

### shRNA probe preparation (half-hairpin barcodes)

To create microarray probe from shRNA pools, a master mixture for each sample containing template DNA (78 k shRNA plasmid pools = 250 pg, 90 k plasmid shRNA pools = 288 pg, genomic DNA = 30 μg), 2x PCR buffer, 2x enhancer solution, 300 μM each dNTP, 900 nM each oligonucleotide primer (PCR_B-fw 5'-Biotin-AATGGACTATCATATGCTTACCGTAACTTGAA-3' and PCR_rev 5'-TGTGGATGAATACTGCCATTTGTCTCGAGGTC-3'), 1 mM MgSO_4_, 45 units of Platinum Pfx polymerase (Invitrogen), and water to 1200 μl was created and divided into 100 μl aliquots. The amplification reaction was performed by denaturing once at 94°C for 5 minutes, followed by (94°C for 15 seconds, 55°C for 15 seconds, 68°C for 20 seconds)x30, 68°C for 5 minutes, then cooling to 4°C.

When electrophoresed on a 2% agarose gel, it is expected that there should be a preponderance of an apparent 178bp PCR product, and as little as possible of an apparent 225bp product. Both bands on the gel represent amplified shRNA barcode sequences, but the upper, slower migrating band is composed of two DNA strands in a cruciform structure, centered around the palindromic shRNA sequence, which is not suitable for further processing into a GMAP probe. DNA in this structure is resistant to restriction enzyme activity at the XhoI restriction site between the shRNA palindrome sequences. Application of PCR product containing a mixture of two apparently different products to a denaturing urea gel resolves a single size species of DNA (data not shown). If PCR reactions are favouring cruciform product, reducing the number of cycles in the PCR reaction will favour the linear product (data not shown). PCR products are immediately purified using the QIAquick PCR purification kit (Qiagen) since long-term storage at 4°C or -20°C enables the conversion of linear product to cruciform DNA. The purified 178bp PCR product is then digested with XhoI (New England Biolabs) for 2 hours at 37°C to generate a thermo-stable half-hairpin probe. The digested samples are separated on 2% agarose gels with lanes large enough to accommodate 150 μl of sample. Using sterile materials, the half-hairpin probes (~106bp) are excised from the gel and purified using a gel extraction kit (QIAGEN). To remove remaining salts, the probe DNA may be passed through a PCR cleanup column, and eluted in a final volume of 30 μl.

### GMAP shRNA probe hybridization

Microarrays were pre-hybridized by one wash with 40°C 10 mM NaOH, followed by incubation at 40°C for 10 minutes with rotation at 40 rpm with a second volume of 10 mM NaOH. The arrays were then slowly washed with 3-5 ml 6x SSPE, 0.0001% Tween 20, followed by filling and incubation for 10 minutes at 40°C with rotation at 40 rpm with 0.0005% Triton X-100. Arrays were then emptied, rinsed twice, then filled with 6x SSPE, 0.0001% Tween 20, and incubated for 2 hours at 40°C with rotation at 40 rpm.

Hybridization solutions consisted of 2 μg of probe for 78 k shRNA pools (2.3 μg for 90 k shRNA pools) in buffer containing 1x MES, 0.89M NaCl, 20 mM EDTA, 0.0001% Tween 20, 0.5 mg/ml BSA, 0.1 mg/ml herring sperm DNA, 0.05 nM biotinylated B2 oligo (Affymetrix), 10% DMSO, 20 μM each blocking oligos (Block_1 5'-AATGGACTATCATATGCTTACCGTAACTTGAA-3', Block_2 5'-TTCAAGTTACGGTAAGCATATGATAGTCCATT-3', Block_3 5'-GTATTTCGATTTCTTGGCTTTATATATCTTGTGGAAAGGACGAAACACCG-3', Block_4 5'-CGGTGTTTCGTCCTTTCCACAAGATATATAAAGCCAAGAAATCGAAATAC-3'), and sterile water to a final volume of 138 μl. Samples in buffer were denatured at 95°C for 10 minutes, incubated at 40°C for 5 minutes, collected by centrifugation then applied to arrays, which were incubated for 16 hours at 40°C at 60 rpm. Arrays were stained with SAPE labeling mix (1x MES staining buffer, 2 mg/ml BSA, 10 μg/ml streptavidin-phycoerythrin), and washed on an Affymetrix fluidics station, then scanned. More complete shRNA probe preparation and hybridization protocols are included in Additional file 2.

### Illumina sequencing

PCR product was prepared as described above for GMAP shRNA probe preparation except that 27 cycles of PCR were employed when amplifying from pooled plasmid DNA. Purified PCR product DNA was used as a template for a second round of amplification in order to incorporate Illumina adapter sequences. Each 100 μl reaction contained 5 ng of template, 2x PCR buffer, 2x enhancer solution, 300 μM each dNTP, 900nM each for Adapter A (5'-AATGATACGGCGACCACCGAAATGGACTATCATATGCTTACCGTAACTTGAA-3') and Adapter B (5'-CAAGCAGAAGACGGCATACGATGTGGATGAATACTGCCATTTGTCTCGAGGTC-3'), 1 mM MgSO_4_, 3.75 units of Platinum Pfx polymerase, and water to 100 μl. The PCR reaction was performed by denaturing at 94°C for 5 minutes, followed by (94°C for 15 seconds, 55°C for 15 seconds, 68°C for 20 seconds)x16, 68°C for 5 minutes, then cooling to 4°C. The resulting 218bp product was purified by electrophoresis in 2% agarose followed by gel extraction. Libraries were initially quantified by the Quant-IT fluorescent assay (Invitrogen), and fragment size was confirmed using a Bioanalyzer high sensitivity chip in a 2100 Bioanalyzer (Agilent Technologies). Libraries were diluted to ~15nM concentration, and concentration was confirmed using commercially available qPCR standard on 1:1000 dilutions of library samples. DNA templates were then diluted to 8pM as per the Illumina cBot user guide, and clusters were generated on a single read flowcell using the Illumina cBot. Each dilution pool was run in a separate lane. Using an Illumina GAIIx instrument, sequence was collected for 22 bases using the primer SeqshRNA (5'-GATTTCTTGGCTTTATATATCTTGTGGAAAGGACGAAACACCG-3').

### Illumina data processing

Raw reads were generated using Illumina's Offline Base Caller software (v1.61). Reads that passed quality filtering were extracted from the QSEQ files and merged into FASTQ files using a custom BASH script. Reads were aligned to a synthetic chromosome, constructed by interspersing the hairpin sense strand sequence with 79nt of random sequence, using MAQ v0.7.1 [32] with default parameters. The synthetic chromosome and hairpin index are available upon request. Aligned sequences were matched to individual hairpin IDs if the alignment start site corresponded with the correct starting nucleotide of the hairpin sequence (i.e.: sequences with mismatches on the first nucleotide were removed) and the MAQ quality score was ≥50. This quality threshold allowed one mismatch over the 21nt sequence. If a read mapped to one of the 227 shRNA barcode sequences duplicated in the shRNA library, it was counted once for each of the duplicate hairpin IDs. Reads were determined in a single replicate experiment, and counts were log_2 _transformed prior to further analysis.

### human ORF probe preparation and hybridization

Human ORFs were amplified from the master hORF pool where each hORF was contained within the pDONR223 plasmid. Five reactions were carried out in a 50 μL volume containing 25 μL of 2x Phusion Flash High-Fidelity Master Mix (Finnzymes), 400nM of each primer and 10 ng of plasmid. The reaction times and temperature were 1 minute at 98°C for 1 cycle; 10 seconds at 98°C, 20 seconds at 60°C, and 4 minutes at 72°C for 30 cycles; 10 minutes at 72°C for 1 cycle. The sequences of the forward and reverse primers specific for the cloned human ORFs were 5'-CACGACGTTGTAAAACGACGGCCAGTC-3' and 5'-GAGCTGCCAGGAAACAGCTATGACCATG-3' respectively. PCR products were purified (Qiagen) and pooled. Purified PCR product was biotinylated (8 reactions, 500ng per reaction) using a BioPrime DNA labelling kit (Invitrogen) and unincorporated biotin-14-dCTP was removed by passing the samples through Sephadex G-50 columns (GE Healthcare). Microarrays were pre-hybridized by adding 130 μL of Hybridization buffer and incubating for 10 minutes at 45°C and 60 rpm. The hybridization mix contained 2x hybridization buffer, 5nM B2 oligo, 50x Denhardt's solution and biotinylated PCR product. Different amounts of labelled probe were added to Affymetrix arrays and hybridized at 45°C for 17 hours with a rotation of 60 rpm. GMAP-UTS520601 Affymetrix arrays were hybridized with 7 μg, 3.5 μg, 1.75 μg, 0.875 μg or 0.4375 μg of biotinylated PCR product obtained from the human ORFeome 5.1 collection. The Human Gene 1.0 ST arrays were hybridized with 3.5 μg of sample. Probe was hybridized to the arrays for 17 hours at 45C and 60 rpm. Arrays were stained with SAPE labelling mix (2x MES staining buffer, 20 mg/mL BSA, and 1 mg/mL streptavidin-phycoerythrin), washed on an Affymetrix fluidics station and scanned.

### GMAP data extraction and processing

Feature signal was extracted from the GMAP and TRCBC arrays using Affymetrix Power Tools v.1.12.0 (APT, http://www.affymetrix.com). The GMAP chip contains triplicate features per shRNA, which we summarized by using the median value. GC-background (GCbg) correction for non-specific probe binding was performed with APT, using feature signals derived from 33,894 GC-background probes on the array. Normalization of replicate arrays was performed with the Bioconductor *affy *package (v1.26.1) in R, using Cyclic Loess based on the '*MA*-plot' of pairs of arrays. Distribution plots generated from the raw, GCbg-corrected, and Loess-normalized signal intensities demonstrated that GCbg-correction served to increase the differentiation between signal from the features corresponding to shRNA pool probes and those features on the GMAP without corresponding partners in the probe pool. while normalization reduced the variance between replicates (Additional file 1, Figure S8).

### Web-based tool for GMAP

We have developed a Java front-end through which this extraction and analysis can be accomplished by users outside our laboratories. The current functionality of the application includes the following:

1. Extraction of signal from a set of Affymetrix. CEL files into a single tab-delimited file. The extracted signal can be limited to a specific subset of features and GC-matched background signal can be subtracted.

2. Signal across chips can be normalized using a variety of procedures. Graphical output is generated for assessment of normalization.

3. Currently, the primary use of this chip is for assessment of strain abundance during pooled growth in a variety of systems that correspond to features on this array. This includes strains with integrated molecular barcodes, transduced with shRNA sequences, or hORF sequences. The Java application includes tools to calculate signal ratios against one or more reference chips with a variety of options available to fine-tune the analysis.

4. Extracted feature signal datasets can be further reduced by limiting to features relevant to specific pools. Standard or user-defined annotation files can be merged with the analyzed signal.

This application, R scripts, links to supporting software and examples of use are available on our supplementary website http://chemogenomics.med.utoronto.ca/supplemental/gmap/.

## List of Abbreviations

APT: Affymetrix Power Tool; DNA: Deoxyribonucleic Acid; GMAP: Gene Modulation Array Platform; HPSI: Hairpin Spike-In; huORF: Human ORF; MGC: Mammalian Genome Collection; ORF: Open Reading Frame; shRNA: Short hairpin RNA; SOP: Standard Operating Procedure; TRC: The RNAi Consortium; TRCBC: The RNAi Consortium Barcode; RNA: Ribonucleic Acid

## Authors' contributions

All authors read and approved the final manuscript. TK developed GMAP shRNA protocols (based on earlier work from EE and KCO), and performed shRNA probe amplification. LEH (along with AS, GSC, SG, RA, TK CN, JM, DER) was responsible for GMAP design. LEH and AS developed Java-based GMAP data extraction tools. LEH assisted with data extraction in shRNA sequencing experiments. DK prepared shRNA probe samples and performed GMAP shRNA pool hybridizations. KRB and JLYK performed provided statistical analysis of shRNA GMAP and sequencing data. RA, AMS, AA performed ORF over-expression experiments and analysis on GMAP. KB and DK provided shRNA reagents. TD performed shRNA sequencing reactions. GSC and XY prepared TRC shRNAs, 90 k shRNA pools and 90 k shRNA infected cell genomic DNA. Experimental work was carried out in the labs of GG, DER, JM and CN. TK and JM wrote the bulk of the manuscript, with contributions from all other authors. JM and CN directed the project.

## Supplementary Material

Additional file 1 **contains additional figures S1-S8 and the corresponding figure legends**. Click here for file

Additional file 2 **contains details methods for GMAP probe generation and hybridization as well as recipes and reagents**. Click here for file
