# Supplementary material for: A comprehensive platform for highly multiplexed mammalian functional genetic screens
Source: BMC Genomics. 2011 May 6;12:213. doi: 10.1186/1471-2164-12-213 (PMC3115879; doi:10.1186/1471-2164-12-213)
Supplement: Additional file 1 — contains additional figures S1-S8 and the corresponding figure legends. [file 1471-2164-12-213-S1.PDF]

Additional Figure Legends

Figure S1 – TRCBC feature offset design for testing signal-to-noise and specificity of shRNA barcode signals. (a) Schematic of feature offset design for TRCBC array. The TRCBC array includes three perfect match (PM) features and a single mismatch (MM) feature corresponding to ~110,000 human and mouse TRC shRNA sequences. PM features 0 and 2 are offset from PM feature 1 by either a 3'C or 5' G, respectively. A reference plasmid pool and dilution series plasmid pool consisting of the same ~45,000 shRNA barcodes were used to generate labeled probe and hybridized separately to TRCBC microarrays. The dilution series plasmid pool contained four subpools of shRNA barcodes that were present at even amounts, diluted 4-fold, 16-fold and 64-fold. Signal distributions were generated for each of the four subpools by subtracting signal intensities from shRNA barcodes in the dilution series subpool from the corresponding shRNA barcodes in the reference subpools and generating 1x, 4x, 16x and 64x distributions for each of PM0 **(b)**, PM1 **(c)** and PM2 **(d)** features. A deconvolution algorithm was designed to measure the fraction shRNAs incorrectly assigned to the proper dilution series pool based on the above data and the results of the assignments are shown on the right of each set of PM distributions, highlighting the observation that more shRNA barcodes are assigned to the correct dilution series pool when considering PM2 feature design.

Figure S2 – Probe amount and hybridization/wash temperature determination. (a) Distribution plots of signal intensity from hybridization of 1μg, 2μg, and 3μg of probe from 78k human shRNA pool. Features on the microarray corresponding to shRNA probe sets in the 78k pool are in solid lines while dashed lines denote remaining shRNA features on the microarray not corresponding to sequences in the probe pool. **(b)** Distribution plots of microarray signal intensity for shRNA probes included in (solid) or excluded from (dashed) the human 78k shRNA pool. 2μg of probe was applied to the GMAP with varying hybridization and wash temperatures as indicated in the legend.

Figure S3 – Readout of high complexity shRNA pools. (a) Binned histograms, and **(b)** cumulative distributions for all 248,049 shRNA features on the GMAP microarray following hybridization of probe generated from the human 78k plasmid shRNA pool or the mouse 78k plasmid shRNA pool. In figures **(a)** and **(b)**, shRNA features that correspond to shRNA barcodes in the plasmid pools are shown as dark red (Human 78k) or dark blue (Mouse 78k) lines while the features that do not correspond to shRNA sequences in the pools (78k-excluded) are indicated as light red or blue lines, respectively. **(c)** Minimum signal for inclusion in analysis is calculated for the human 78k Even pool as mean of background plus 1.96 standard deviations (95% confidence limit).

Figure S4 – Comparison of TRCBC and GMAP chips. Scatter plot depicting the signal intensities for probe generated from genomic DNA from breast cancer cells (BT-474) infected with the human 54K shRNA pool hybridized to TRCBC and GMAP arrays (Pearson's correlation coefficient R=0.96). The amount of probe hybridized to the TRCBC array was 2μg and the GMAP array was 1.35μg.

Figure S5 – Distribution plots for GMAP and deep sequencing data. Distributions for **(a)** deep sequencing read number, and **(b)** GMAP signal intensity of the undiluted shRNAs present in the Even, 4x, 16x and 64x pools.

Figure S6 – HSPI features on the GMAP. (a) Images of GMAP chips following hybridization of probe generated from the human 78k plasmid pool in the absence of spike-in controls (left image) and the presence of 12 spike in probes (5nM) selected to represent the range of shRNA Tm's. **(b)** Quantification of signal for 12 selected spike-in features averaged over 25 HSPI replicates. Final concentrations of each HSPI oligonucleotide tested in the hybridization mixtures were 0pM, 50pM, 500pM and 5nM.

Figure S7 – Comparison of 90k Plasmid and Genomic probe data. Average shRNA feature intensity data from triplicate template amplifications and GMAP hybridizations for probe generated from shRNA plasmid template and shRNA-containing A549 genomic DNA is highly correlated (Pearson correlation coefficient R=0.971).

Figure S8 – Distribution plots of data from triplicate GMAP hybridizations using probe containing the human 78k Even pool. Plots include raw data, data after GC-background (GCbg) correction, and data post-Cyclic Loess normalization.

Figure S1

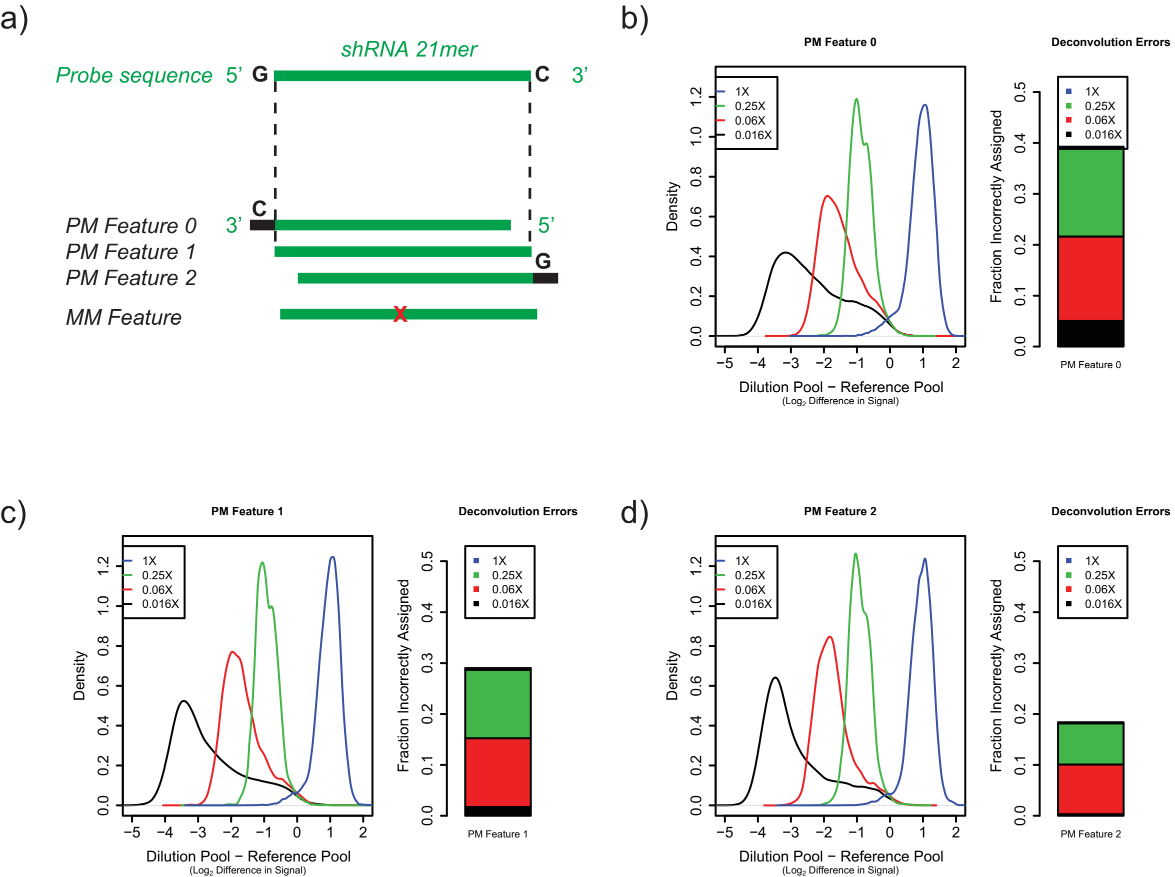

a)
shRNA 21mer
Probe sequence 5' G
C 3'
C
PM Feature 0 3'
5'
PM Feature 1
G
PM Feature 2
MM Feature
b)
PM Feature 0
Deconvolution Errors
1X
0.25X
0.06X
0.016X
Density
Dilution Pool − Reference Pool
(Log2 Difference in Signal)
Fraction Incorrectly Assigned
PM Feature 0
c)
PM Feature 1
Deconvolution Errors
1X
0.25X
0.06X
0.016X
Density
Dilution Pool − Reference Pool
(Log2 Difference in Signal)
Fraction Incorrectly Assigned
PM Feature 1
d)
PM Feature 2
Deconvolution Errors
1X
0.25X
0.06X
0.016X
Density
Dilution Pool − Reference Pool
(Log2 Difference in Signal)
Fraction Incorrectly Assigned
PM Feature 2

Figure S2

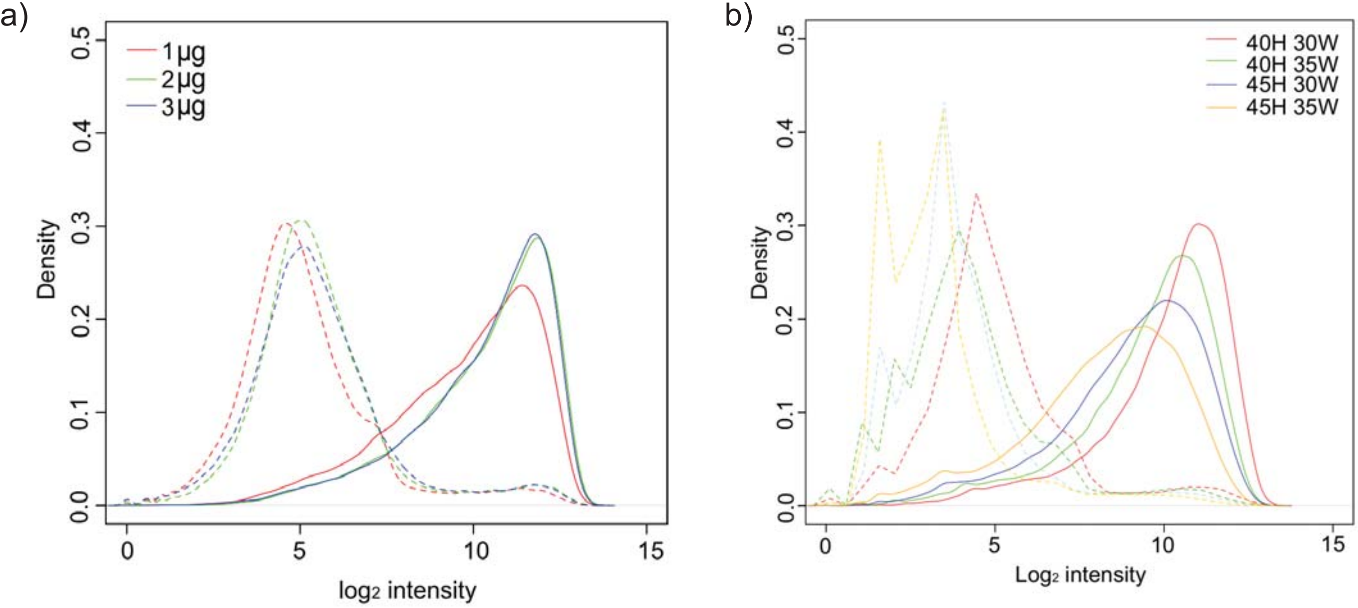
a)
1 µg
2 µg
3 µg
Density
log2 intensity
b)
40H 30W
40H 35W
45H 30W
45H 35W
Density
Log2 intensity

Figure S3

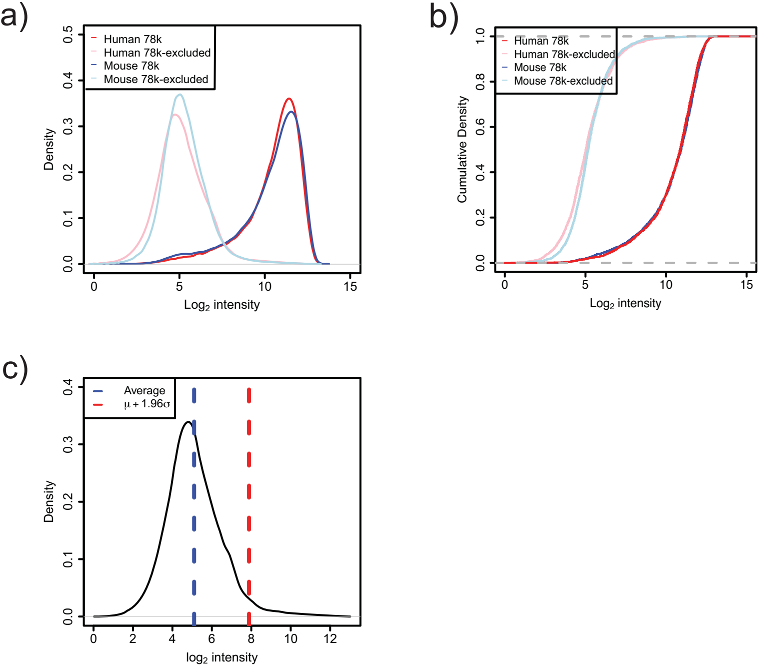
a)
Human 78k
Human 78k-excluded
Mouse 78k
Mouse 78k-excluded
Density
0.0
0.1
0.2
0.3
0.4
0.5
0
5
10
15
Log2 intensity
b)
Human 78k
Human 78k-excluded
Mouse 78k
Mouse 78k-excluded
Cumulative Density
0.0
0.2
0.4
0.6
0.8
1.0
0
5
10
15
Log2 intensity
c)
Average
μ + 1.96σ
Density
0.0
0.1
0.2
0.3
0.4
0
2
4
6
8
10
12
log2 intensity

Figure S4

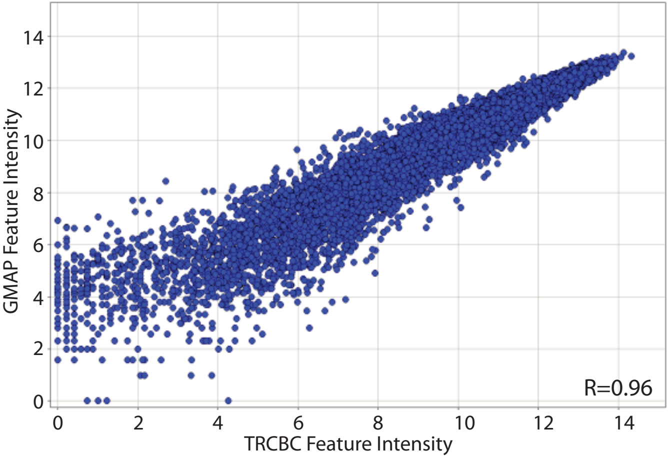

GMAP Feature Intensity
TRCBC Feature Intensity
R=0.96
0
2
4
6
8
10
12
14

Figure S5

a)

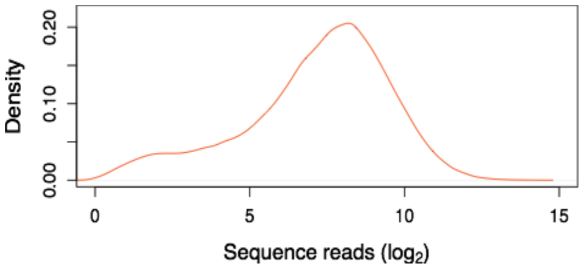
Density
0.00
0.10
0.20
0
5
10
15
Sequence reads (log_2)

b)

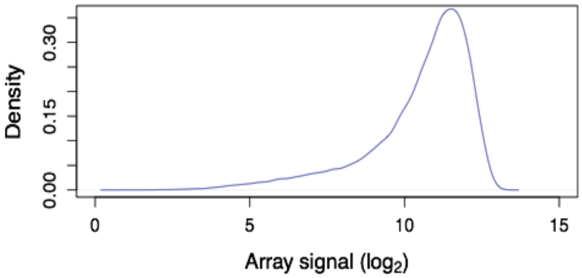
Density
0.00
0.15
0.30
0
5
10
15
Array signal (log_2)

Figure S6

a)

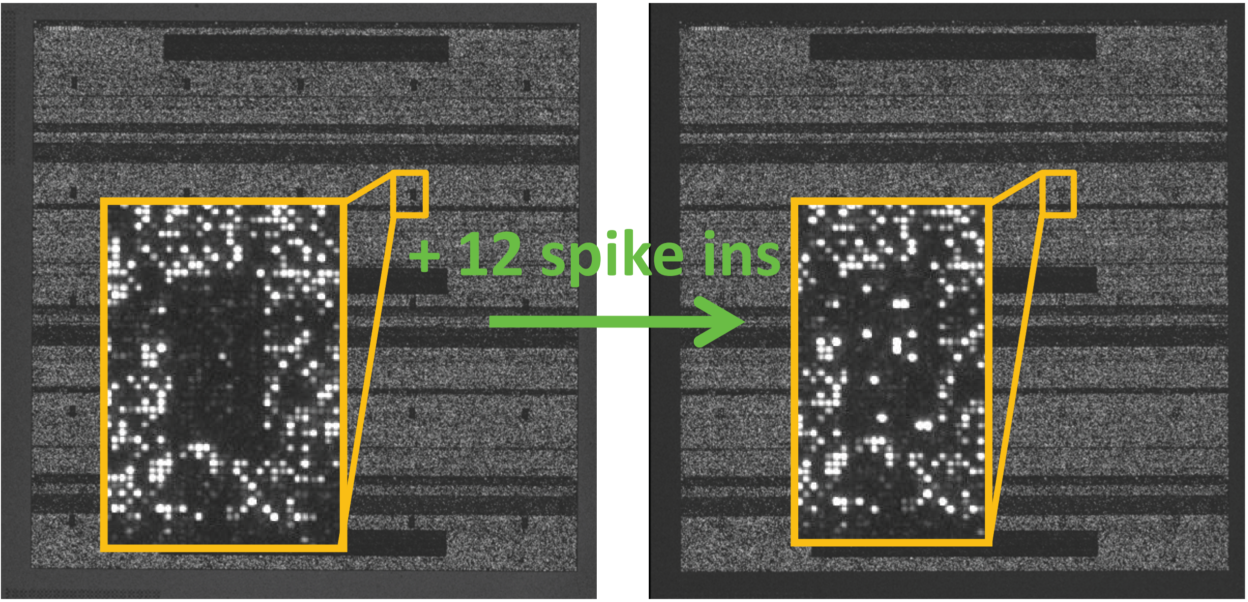
+ 12 spike ins

b)

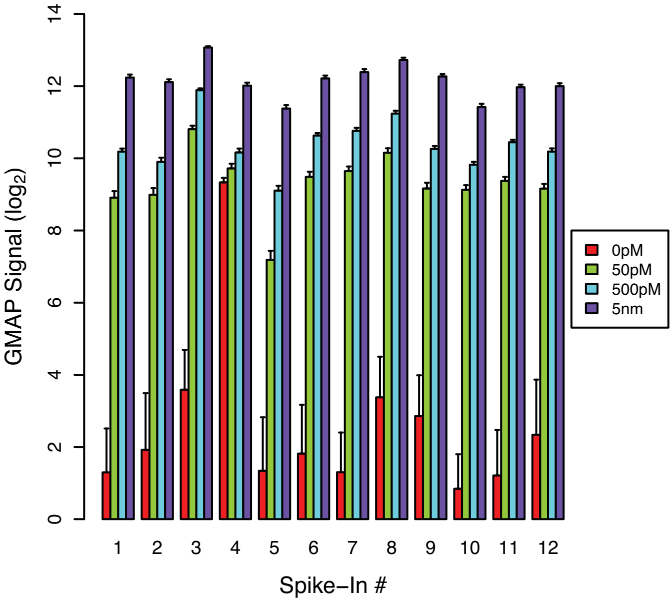
GMAP Signal (log_2)
0
2
4
6
8
10
12
14
1
2
3
4
5
6
7
8
9
10
11
12
Spike−In #
0pM
50pM
500pM
5nm

Figure S7

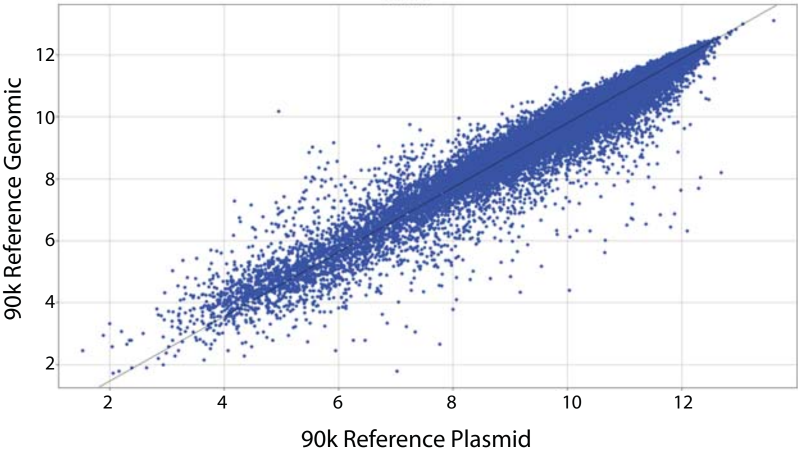

Figure S8

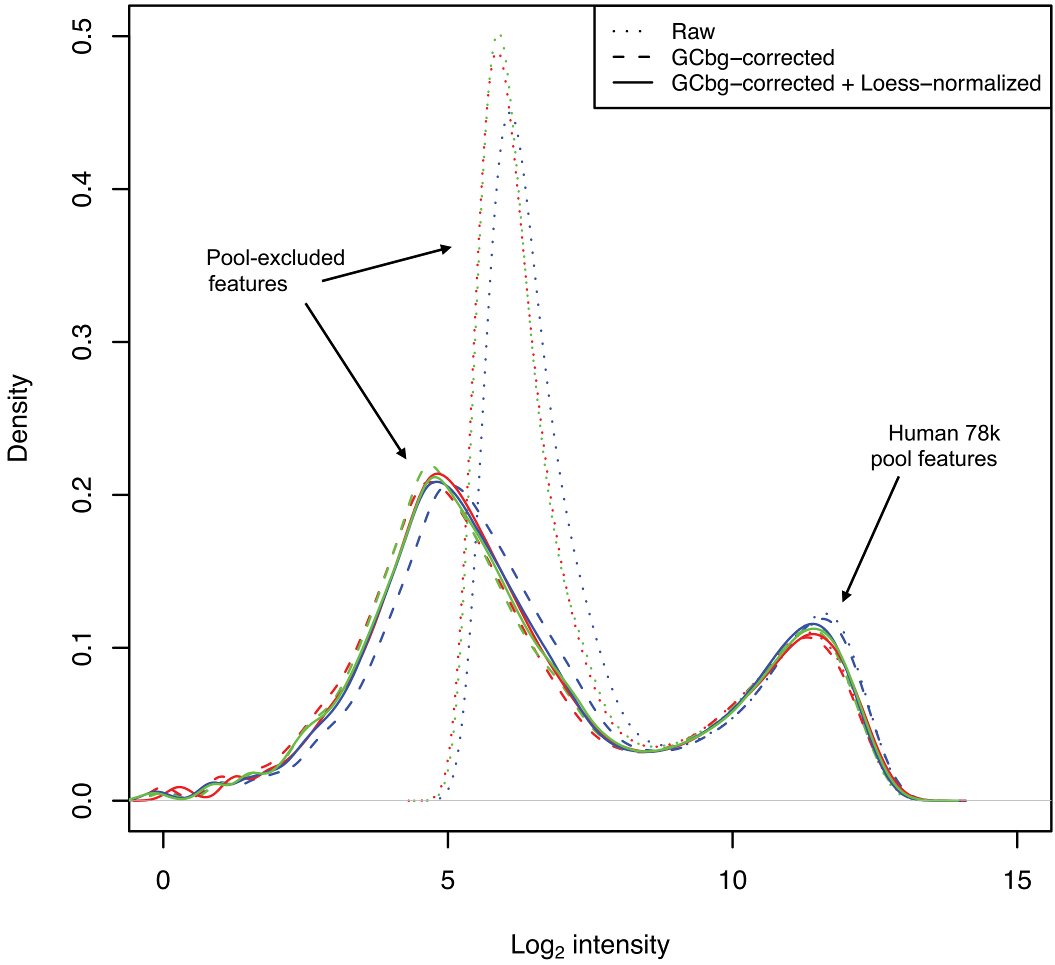

Raw
GCbg−corrected
GCbg−corrected + Loess−normalized
Pool-excluded
features
Human 78k
pool features
Density
Log2 intensity
0.0
0.1
0.2
0.3
0.4
0.5
0
5
10
15
