# Supplementary material for: A comprehensive platform for highly multiplexed mammalian functional genetic screens
Source: BMC Genomics. 2011 May 6;12:213. doi: 10.1186/1471-2164-12-213 (PMC3115879; doi:10.1186/1471-2164-12-213)
Supplement: Additional file 2 — contains details methods for GMAP probe generation and hybridization as well as recipes and reagents. [file 1471-2164-12-213-S2.PDF]

Additional Information includes:

1) **Detailed methods for probe generation and hybridization (Pages 2-7)**
2) **Recipes and reagents (Pages 8-10)**

Additional Methods

Probe Amplification

Note: In order to have sufficient representation of the shRNA sequences from the experimental cell population, a large quantity of gDNA (20-30µg) must be used as template for probe amplification.

Note: Use plugged tips for **ALL** PCR setup steps. Unplugged tips may be used for all steps after amplification.

- Per sample, add the reagents below to a sterile microcentrifuge tube.
- Remove all buffers from the area of PCR set-up before opening of gDNA sample tubes.
- Add gDNA to each PCR mixture, mix samples again by repeat pipetting 500µl 10 times, then close the microcentrifuge tubes until samples are aliquoted into PCR tubes.
- Aliquot 99µl into each tube on a strip of 8 (54k) or 12 (80k) tubes.

PCR Recipe

	54k pool	80k pool	-ve control
10x PCR amplification buffer	160µl	240µl	20µl
10x enhancer buffer	160µl	240µl	20µl
10mM dNTP mix	24µl	36µl	3µl
20uM primer mix	36µl	54µl	4.5µl
50mM $MgSO_4$	16µl	24µl	2µl
Water (392µl-Xµl gDNA)	392µl-Xµl	588µl-Xµl	49µl
PFX enzyme	12µl	18µl	1.5µl
gDNA template	Xµl (20µg)	Xµl (30µg)	**MIX**
	**MIX**	**MIX**	100 µl
	800µl	1200µl	

Amplification Program

Step	Temp	Time (min)
1	94°C	5:00
2	94°C	0:15
3	55°C	0:15
4	68°C	0:20
5	Go To Step 2 (29X)	
6	68°C	5:00
7	4°C	hold

- After program finishes, **do not** store the product at either 4°C or -20°C as this enables the conversion of double-stranded product to restriction digest-resistant cruciform DNA. Instead, immediately purify the PCR-products using the QIAquick PCR purification kit and the modified protocol below.
- When run on a 2% agarose gel, the PCR product should have a pre-dominance of ~178bp product (example A below), and as little as possible of the ~225bp band. Both bands actually contain the same sequence of bases, but the larger band is composed of two DNA strands in a cruciform structure, centered around the palidromic shRNA

sequence. PCR product in this form is resistant to restriction digest at the XHOI site in the middle of the shRNA palindrome since the DNA is not double stranded in this area. If the cruciform product predominates (as in example B below), try reducing the total number of PCR cycles.

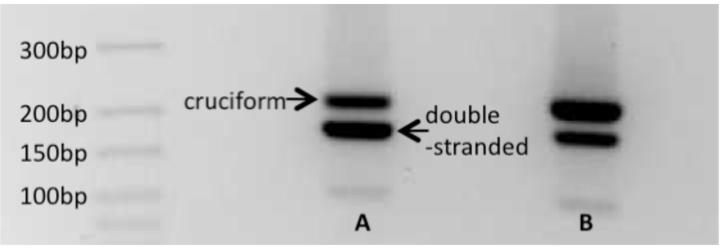

Probe Purification

Quantities are described for **54k** or **(80k)** PCR purification

- Combine the **8** or **(12)**x99µl PCR reactions in a 15-ml screw-cap tube, and remove 10µl to run on a gel.
- Add **4** **(6)** ml Qiagen buffer PB to the sample and vortex to mix.
- The sample will be split onto **2** **(3)** columns, using half the sample per column:
- Load 750µl of sample at a time on each column and centrifuge for 60 seconds at 13,000rpm.
- Discard flow-through and repeat until all the sample has been passed through the columns.
- To wash, add 750µl Qiagen buffer PE to the column and centrifuge for 60 seconds at 13,000 rpm
- Aspirate flow-through and place the column back in the same tube.
- Centrifuge for 60 seconds at 13,000 rpm to dry the column.
- Place each column in a clean 1.5ml microcentrifuge tube.

For 54k PCR samples

- Add 30µl elution buffer EB to the centre of the membrane on each column. Let sit for 1 minute then centrifuge for 1 min at 13,000 rpm.
- Repeat elution step with another 30µl of EB and combine elutions from the 2 columns of the same tube.
- Set aside 1.5µl of the approximately 110µl total volume of purified PCR-sample to check on a gel.

For 80k PCR samples

- Add 40µl elution buffer EB to the centre of the membrane on each column. Let sit for 1 minute then centrifuge for 1 min at 13,000 rpm.
- Set aside 1µl of the approximately 110µl total volume of purified PCR-sample to check on a gel.

- Measure DNA concentration of the sample.
- When run on a 2% agarose gel, the pre and post-column purification samples should contain PCR product bands of roughly equal intensity, and the oligos should be absent in the post-column samples (see below).

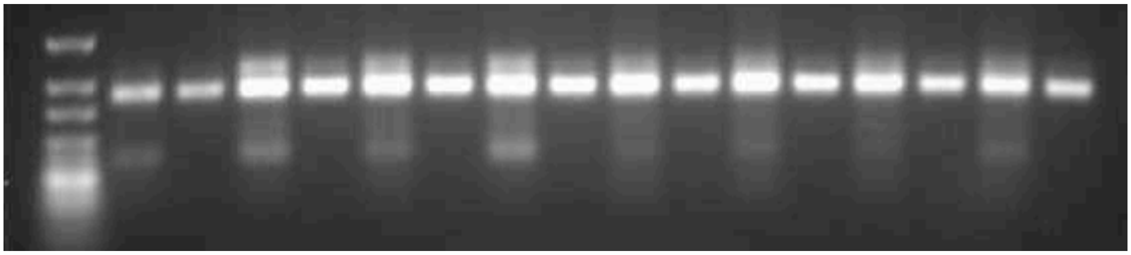

Restriction Digest of PCR Product

- Set up restriction digest reaction:

Purified PCR-product	105μl
10x NEB buffer 2	12μl
10mg/ml BSA	1.2μl
XHOI (10,000U/ml)	2μl
	120.2μl

- Mix contents well by pipetting, then divide into 2x 60μl in PCR tubes.
- Incubate for 2 hours at 37°C in thermal cycler. Stop reaction by heating at 65°C for 20 min, then cooling to 4°C. Once it has been cut, it is safe to leave the sample at 4°C for extended periods if necessary.

Gel Electrophoresis and Purification of Probe

- Prepare 2% agarose gel(s) with lanes large enough to accommodate 150μl. Wide and thin lanes preferred over thick lanes so that the bands are compact, not smeared.
- Combine 2x60μl of digested sample and add loading dye, mix.
- Run the gel at 120V for 45 minutes.
- Take a quick picture of the gel for documentation, leaving the gel on the tray to minimizing UV exposure.
- Using fresh razor blades, cut out the probe for each sample. The brightest, middle band, running at ~100bp contains the desired probe DNA. Trim as much excess agarose around the band as possible.

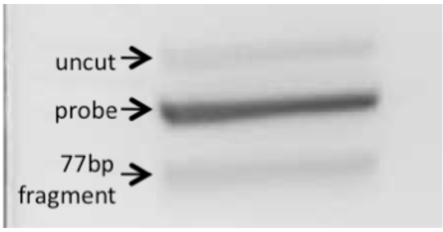

- Transfer the gel slices to pre-weighed 15ml screw-cap tubes .
- Weigh the tubes containing gel slices and calculate the weight of the gel slices (should be between 300-400mg).
- Add 3 gel volumes of buffer QG (eg. 300μl per 100mg).
- Incubate at 50°C for 15-20 minutes, vortexing every 2-3 minutes.

- To ensure complete dissolution of the agarose, the samples should remain in the water bath for several minutes after it has visibly dissolved.
- Add 1 gel volume of isopropanol to the sample and vortex.
- 400mg is the maximum gel weight per column, divide samples if your slice(s) weighed more.
- Add 750µl sample to each column and centrifuge 1 minute at 13,000rpm.
- Discard flow-through and repeat until all the sample has been passed through the columns.
- Add 750µl buffer PE. Incubate for 5 minutes at room temperature, then centrifuge 1 minute at 13,000rpm.
- Aspirate the flow-through to remove all of the PE buffer.
- Centrifuge tubes at 13,000 rpm for 1 minute, to dry the column.
- Put columns in clean 1.5ml tubes.
- Add 30µl EB to each column, leave at room temperature for 1 minute then centrifuge for 1 minute at 13,000rpm.
- Repeat elution step with another 30µl of EB and combine elutions from the 2 columns of the same sample (~60µl).
- Check the concentration and purity on a spectrophotometer.
- Expect the 260/280 ratio to be 1.8-2.0, and the 260/230 to be 0.2-0.4.
- Use the Qiagen PCR purification kit to clean the samples and remove more of the salts (remaining from the QG buffer).
- Use one column per sample, do one PE wash with a 5 minute incubation prior to centrifuging.
- Elute 2x30µl with EB, incubating for 1minute at RT before centrifuging.
- Check the concentration and purity on a spectrophotometer.
- Expect the 260/280 ratio to be 1.8-2.0, and the 260/230 to be 1.7-2.1.
- You should have over 2µg (average yield is 3-3.5µg) of each sample to hybridize on chips.

<u>Pre-conditioning of chips</u>

- Let chips warm up to room temperature on bench for about an hour.
- Fill the chip slowly with 40°C 10mM NaOH, then remove the liquid.
- Fill the chip again with 40°C 10mM NaOH. Incubate with rotation in hybridization oven for 10 minutes at 40°C, 40rpm.
- Cut the head off of a 200µl pipette tip and fit the tip onto a 5ml-syringe.
- Rinse chip slowly with 3-5mL wash buffer A using the 5ml syringe, collecting the flow-through in a beaker.
- Remove wash Buffer A, then fill the array with 0.0005% Triton and incubate in hybridization oven for 10 min at 40°C, 40rpm.
- Empty the chip, then rinse it 2x with wash buffer A by filling and emptying the chip.
- Fill up chip with wash buffer A.
- Put chips in the hybridization oven at 40°C, 40-60 rpm for 2-4hrs.

Hybridization Mix preparation

- The mixture of blocking oligos is made up of **block_1**, **block_2**, **block_3 and block_4**, each at a concentration of 20µM.
- Aliquots of ~105µl (4 chips worth) should be prepared to reduce the number of freeze/thaw cycles on stocks.
- Prepare the probe mixture in a microcentrifuge tube.

	54k pool-GMAP chips	80k pool – GMAP chips
2x hybridization buffer	66µl	66µl
50mg/ml BSA	1.3µl	1.3µl
10mg/ml herring sperm DNA	1.3µl	1.3µl
5nM B213	1.3µl	1.3µl
Blocking oligo mix	16.9µl	25µl
Spike-in oligo mixture	1.4µl	1.4µl
DMSO	13.2µl	13.2µl
Sample (2µg)	Xµl	Xµl
Water	36.6µl-Xµl	28.5-Xµl
	138µl	138µl

- Incubate the tube in a boiling water bath for 10 minutes.
- Transfer sample to a 40°C water bath for 5 minutes.
- Centrifuge tubes at 13,000 rpm for 5 minutes.
- Remove wash A from the chips and fill them with the sample solution, avoiding any debris in the bottom of the tube.
- Cover the ports on the chips with tough-spot seals.
- Incubate the chips for 15-16 hours at 40°C, 60 rpm.

Chip staining and washing

Prime the fluidics station:

- Put all tubing in place, empty waste bottle, fill wash A and B bottles, fill up Millipore water bottle.
- Use fluidics station protocol “PRIME_450”.
- Prepare **SAPE labeling mix** and make 2 aliquots of 590µl each per chip.
- Prepare **antibody labeling mix** and make 1 aliquot of 590µl per chip.

SAPE labeling mix (per chip)	
2x MES stain buffer	600µl
50 mg/ml BSA	48µl
1 mg/ml SAPE	12µl
High quality water	540µl
	1200µl

Antibody labeling mix (per chip)

2x MES stain buffer	300µl
50 mg/ml BSA	24µl
30 mg/ml IgG	2µl
500 µg/ml BAS*	3.6µl
High quality water	270.4µl
	600µl

- Aliquot 590µl/chip for each solution into microcentrifuge tubes.
- Remove tough-spots from chips.
- Remove hybridization mix (~2x150µl) and set aside for -20°C storage for potential future use.
- Fill chip with wash A.
- On scanner computer, under "Experiments", scan the barcode and type in the sample information.
- Use the protocol "FlexGE_WS2v5_450_KCEE" (runtime is ~75 min).
- When the chips are ready, check for air bubbles and wash again if necessary.
- Put tough spots on the chips and scan them.
- When done with the fluidics, put all tubing in Millipore water and run "SHUTDOWN_450" program.
- When shutdown and scanning is finished, release all fluidics tubing and shut off stations and laser.

Recipes and Reagents

Enzyme used for probe amplification is Platinum *Pfx* DNA polymerase (Invitrogen)

B213 (biotinylated control oligo): 5'-***B***-CTGAACGGTAGCATCTTGAC-3'

Prepare 5nM **B213** solution by serial dilutions:

- Dissolve dry oligo at 100μM and dilute 1:100 (10μl 100μM primer in 990μl sterile 10mM Tris pH7.5), mix very well to obtain 1μM.
- Do another 1:100 dilution (10μl of 1uM primer in 990μl sterile 10mM Tris pH7.5), mix very well to obtain 10nM.
- Dilute 1:2 (100μl 10nM in 100μl sterile 10mM Tris pH7.5) to obtain 5nM.

PCR_B-fw: 5'-***B***-AATGGACTATCATATGCTTACCGTAACTTGAA-3'
PCR_rev: 5'-TGTGGATGAATACTGCCATTTGTCTCGAGGTC-3'

Prepare mixed stock of **PCR_B-fw** and **PCR_rev,** each at 20μM in sterile 10mM Tris pH7.5, and split into aliquots for frozen storage.

Blocking oligos:

Block_1 5'-AATGGACTATCATATGCTTACCGTAACTTGAA-3'
Block_2 5'-TTACCTGATAGTATACGAATGGCATTGAACTT-3'
Block_3 5'-GTATTTCGATTTCTTGGCTTTATATATCTTGTGGAAAGGACGAAACACCG-3'
Block_4 5'-CGGTGTTTCGTCCTTTCCACAAGATATATAAAGCCAAGAAATCGAAATAC-3'

To prepare 1000μl of blocking oligo solution with each oligo at a final concentration of 20μM:

- Dissolve dry oligos at 200μM in sterile 10mM Tris pH7.5.
- Add 100μl of each oligo stock to 600μl of sterile 10mM Tris pH7.5, mix well.

WASH A (Non-stringent wash buffer)

20XSSPE	300ml
10% Tween	1ml
Milli-Q water	699ml
	1000ml

WASH B (Stringent wash buffer)

12x MES stock	83.3ml
5M NaCl	5.2ml
10% Tween20	1ml
Milli-Q water	910.5ml
	1000ml

Filter **Wash B** and store at 4°C wrapped in foil.

12X MES

MES free acid monohydrate	7.04 g
MES Sodium Salt	19.3 g
Molecular Biology Grade water	80 ml

Mix well for >10 min on stirrer, pH to 6.5-6.7,
Adjust volume to 100ml.
Pass through a 0.2µm filter and store at 4°C, shielded from light.
Solution can be stored up to one year. Changes colour when it goes bad.

2X Hybridization Buffer

12X MES Stock	8.3 ml
5M NaCl	17.7 ml
0.5M EDTA	4.0 ml
10% Tween 20	0.1 ml
Milli-Q water	19.9 ml
	50.0ml

Pass through a 0.2µm filter and store at 4°C, shielded from light.
Solution can be stored up to one year. Changes colour when it goes bad.

2X MES stain buffer

12X MES Stock	8.3 ml
5M NaCl	18.5 ml
10% Tween 20	1 ml
Milli-Q water	22.2ml
	50.0ml

Pass through a 0.2µm filter and store at 4°C, shielded from light.
Solution can be stored up to one year. Changes colour when it is bad.

IgG antibody
Dissolve 30mg IgG/ml in sterile, filtered 150mM NaCl. OK to heat to 55°C and vortex slightly.
Mix well, looks blurry but OK.
Make 25µl-aliqouts, and store at 4°C.

Biotinylated anti-streptavidin phycoerythricin preparation ("BAS")
Reconstitute by adding 1ml Molecular Biology Grade water to 0.5mg antibody.
Mix well and make 25ul-aliqouts. Store at 4°C.

BSA
Dissolve 50mg Bovine Serum Albumin/ml in Molecular Biology Grade water.
Filter-sterilize and aliquot (make 1ml and 50ul-aliqouts). Store at -20°C.

Spike-in Oligo Stock
A stock mixture of the following twelve 5'-biotinylated oligos, each at 5nM in 10 mM Tris pH7.6 was used as the source of spike-in controls.

5'-B-GTAAATCTAGTGCCACCAAGTC-3'
5'-B-TCCCTTTGTTCCTTAGTCATCC-3'

5’-B-AGATCTAAAGCATGAGGGTCGC-3’
5’-B-ACCACCACAATATGCATGTGAC-3’
5’-B-TGTCATAACCTCCTACCCATCC-3’
5’-B-TTTGAATCTCTGGTCAGAAGCC-3’
5’-B-ATGGCAATACATAGGTGTGAGC-3’
5’-B-TAATGAGATTGACCAATGGCCC-3’
5’-B-ACTACCTCTAGACACCAGTGAC-3’
5’-B-CAGGTTCAACCTTAAAGTGCAC-3’
5’-B-GGAATCAAACTAGACAGATGGC-3’
